# The Medical Curriculum

**Published:** 1923-09

**Authors:** 


### THE MEDICAL CURRICULUM.

### THE ENGLISH CONJOINT COURSE.

The student who desires to take the Conjoint qualification—that is to say, the double diploma of Member of the Royal College of Surgeons of England and Licentiate of the Royal College of Physicians of London—must comply with the regulations laid down for medical qualifications generally. Students are required to pass a pre-medical examination in chemistry and physics conducted by the Conjoint Examining Board before commencing the five years' curriculum of professional study or some other examination recognised by the Board—namely, the examination in chemistry and physics for the Degree in Medicine of any University recognised by the Board; the Higher School Certificates of Oxford and Cambridge Universities, and of the Oxford and Cambridge Schools Examination Board; the Higher Certificates of London, Bristol, Durham Universities, and of the Joint Matriculation Board of the Northern Universities. A candidate must enter for Chemistry and Physics together, and he will not be allowed to pass in one without obtaining at the same time at least half the number of marks required to pass in the other subject. He will be admitted to the examination on producing evidence of having passed the required Preliminary Examination in General Education and of having received instruction during 180 hours in Chemistry and 120 hours in Physics to the satisfaction of his teachers. These courses may be commenced or attended before the required Preliminary Examination in General Education is passed. The examination is partly written, partly oral and partly practical. A candidate rejected in one or both subjects of the examination will not be admitted to re-examination until after the lapse of a period of not less than three months, and he must produce evidence of further instruction in the subject or subjects of failure. The fee for the examination is three guineas, for re-examination in Chemistry two guineas, and for re-examination in Physics one guinea.

### The Professional Examination.

There are two professional examinations called the First and Final Examinations. The courses of study for these examinations must not be commenced until the pre-medical examination in Chemistry and Physics or some equivalent examination has been passed. The subjects of the First Professional Examination are:—Section I.: (*a*) Anatomy, including histology and embryology; (*b*) Physiology, including bio-chemistry. Section II.: Pharmacology, practical pharmacy and materia medica. A candidate must have attended at a recognised medical school courses of instruction in anatomy, including embryology, during five terms, during which he must have dissected the whole body, courses of instruction in physiology, including general biology, bio-chemistry and bio-physics, during five terms; courses of instruction in pharmacology, practical pharmacy and materia medica. A candidate may present himself for the two sections together or separately, but he must take parts (*a*) and (*b*) of Section I. together until he has passed in one or both parts, but a candidate will not be allowed to pass in one part unless he obtains at the same time at least half the number of marks required to pass in the other part. Section II. of the examination may be passed at any time before the candidate enters for the Final Professional Examination. The fee for the First Professional Examination is ten guineas, for re-examination after rejection in Section I. six guineas, for re-examination after rejection in either part of Section I. three guineas, for re-examination after rejection in Section II. three guineas. A candidate who produces satisfactory evidence of having passed an examination in the subjects of Section I. or of either part of Section I. and of Section II. in the examination for the Degree in Medicine conducted at a University recognised by the Board will be exempted from further examination in such subject or subjects.

### The Final Examination.

The subjects of the Final Professional Examination are:—Section I.: (*a*) Pathology, including morbid anatomy, morbid histology and clinical pathology; (*b*) Bacteriology. Section II., Part 1: Medicine, including medical anatomy, forensic medicine and public health. Part 2: Surgery, including surgical anatomy and the use of surgical appliances. Part 3: Midwifery and gynæcology. The examination is partly written, partly practical, partly clinical and partly oral. A candidate may take Sections I. and II. and the three parts of Section II. of the Final Examination separately, or may take the whole examination together. He will be required to produce the certificates required by the regulations before being admitted to the respective parts of the examination. A candidate who produces evidence of having passed an examination for a Degree in Medicine in the subjects of Pathology and Bacteriology at a University recognised by the Board is exempted from Section I. The fee for admission to Section I. is four guineas; for admission to Section II., Part 1, ten guineas; Part 2, ten guineas; Part 3, six guineas; and the re-examination fees are respectively three guineas, six guineas, six guineas and four guineas.

### Special Diplomas.

The Royal Colleges also grant special Diplomas in Public Health, Tropical Medicine and Hygiene, Ophthalmic Medicine and Surgery, and in Psychological Medicine, particulars of which can be obtained from the Secretary of the Conjoint Board, Examination Hall, 8-11 Queen Square, Bloomsbury, London, W.C. 1.

### LONDON UNIVERSITY COURSE.

The University of London confers the degrees of Bachelor of Medicine and Bachelor of Surgery (M.B., B.S.), Doctor of Medicine (M.D.), and Master of Surgery (M.S.). The Senate has also recently established a Bachelor's Degree in Dental Surgery (B.D.S.). A student who desires to take the degrees of M.B., B.S., or the degree of B.D.S., must first be registered as a matriculated student either by passing the matriculation examination in one of its forms or by being exempted from the examination on the ground of having passed an approved examination of equal standard in another University. The course normally extends over 5½ years from matriculation, and the whole course may, and the greater part must, be taken at one or more of the medical institutions recognised by the University. It is not necessary for a student to apply for registration as a medical student by the General Medical Council in order to qualify for the examinations in medicine of the University.

### The First and Second Examinations.

In the first year of the medical course inorganic chemistry, physics, and general biology in preparation for the first examination for medical degrees are taken, the examination being held in July and December. Organic (including Bio-) Chemistry is studied in the first half of the second year for Part I. of the second examination, which is taken normally six months after passing the first examination. Part I. of the second examination is held in March and July. The second and third years are devoted to organic (including Bio-) Chemistry, human anatomy and embryology, physiology, and pharmacology. The second medical examination, Part II., is normally taken 2½ years after matriculation, the examinations being held in March and July.

### The Final Course for the M.B. and B.S.

In the final course for the M.B., B.S. (the two degrees must be taken together), the subjects are medicine, surgery, midwifery and diseases of women, pathology, forensic medicine, and hygiene. The examination may be taken in two groups, and is held in May and October. The examination is written, clinical, oral, and practical. The candidate is required to examine and report on selected cases, to do practical pathological work, and to be acquainted with the essentials of operative surgery, though actual operations on the dead subject are not demanded. Exemption may be granted in certain subjects at the first and second medical examinations to students who have passed equivalent examinations of the University in the same subjects.

### Dental Qualification.

The course of study for the degree of B.D.S. extends over five years from matriculation, and a student must pass in succession the First, Second, Third, and Fourth Examinations in Dental Surgery. Certain exemptions may be granted to those who have passed equivalent examinations of the University in the same subjects. The detailed regulations in the Faculty of Medicine (including those relating to the Bachelor's Degree in Dental Surgery) for internal students may be obtained from the Academic Registrar, University of London, South Kensington, S.W. 7.

### OXFORD UNIVERSITY COURSE.

At Oxford University two degrees in medicine can be obtained, the B.M. and the D.M.; similarly in surgery there are two degrees, the B.Ch. and the M.Ch. No candidate is eligible for any of these degrees unless he is a graduate in Arts, that is to say, either an M.A. or a B.A.; in which branch of the Arts that degree has been obtained is a matter of little moment. The usual course of the medical student who has passed Reponsions is to work at the Preliminary Science examinations in physics, chemistry, and zoology and botany. A candidate who has passed or who is qualified for exemption from Responsions is permitted to enter his name for the Preliminary examinations before matriculation, and after passing them, to spend a further two years in study for the Final Honour School of Physiology, in which he takes his degree of B.A. He next has to pass the first B.M. examination in organic chemistry in relation to medicine, in human anatomy, and (unless he has obtained a first or second class in the Honour School of Physiology) in human physiology. Some candidates, however, take the First B.M. before their Final School. After this he prepares for the second B.M. examination, which comprises the subjects of materia medica and pharmacology, pathology, forensic medicine and hygiene, and the Final subjects of medicine, surgery, and midwifery. When the examinations in these subjects have been passed, the candidate may supplicate for and obtain the degree of B.M., B.Ch.

### The D.M. and the M.Ch.

The degree of D.M. is granted to Bachelors of Medicine of the University who have entered their thirtieth term—it should be remembered that there are three terms in the academical year—who have presented a dissertation on some medical subject that is approved by the Board of the Faculty of Medicine. The degree of M.Ch. is granted to Bachelors of Surgery of the University who have entered their twenty-first term from matriculation and satisfied some other requirements; the examination is held annually in June. For further information regarding the School of Medicine application may be made to the Dean, Department of Medicine, Museum, Oxford.

### CAMBRIDGE UNIVERSITY COURSE.

The degrees of Bachelor of Medicine (M.B.) and Bachelor of Surgery (B.Ch.) at this University are only conferred after the entire course of study with examinations for both has been carried out. The B.Ch. is, therefore, in itself a complete and registrable qualification to practice, and is frequently so used. To obtain either of these degrees it is necessary to pass five years in the study of medicine after registration as a student, and to reside for three years at the University. The professional examinations are three in number. The first comprises general and inorganic chemistry, mechanics, physics, biology; like similar examinations elsewhere, it is divided into three parts, which may be passed separately. The second is divided into three parts: Part I. Organic chemistry; Part II. comprising human anatomy and physiology; Part III. elementary pharmacology and pharmaceutical chemistry and general pathology. The third is also divided into two parts—Part I. including surgery and midwifery, Part II. physics and pathology and pharmacology. When the third examination has been completed the student may take the degree of B.Ch. at once, but for the M.B. he must prepare and submit a thesis on some subject in medicine, surgery, or midwifery, selected by himself.

### The Advanced Degree in Surgery.

For the degree of M.D. it is necessary to have been M.B. at least three years, or else M.A. for at least four years, having passed all the examinations required for B.Ch., and to submit a thesis on some subject in medicine (not in surgery) which is a definite research or contribution to the knowledge of its subject. There is also an advanced degree in surgery (Master of Surgery, M.Ch.) which is said to demand a distinctly higher standard of surgical knowledge and proficiency than the English F.R.C.S. Not many candidates attempt it, and fewer still attain it; practically all who possess it are surgeons on the staff of important general hospitals.

### LONDON MEDICAL SCHOOLS.

### University College: University of London.

The College Faculty of Medical Sciences comprises the departments of physics, chemistry, botany, and zoology (the preliminary medical sciences), also the departments of anatomy, physiology, and pharmacology (the intermediate medical sciences), and the department of hygiene and public health (post-graduate study). Research is undertaken in all departments of study. Composition fees: For the courses required by the University of London—(1) For the medical course, 35 guineas. (2) For the second medical course, 80 guineas, payable in two instalments of 40 guineas each. For the medical education required by the Examination Board in England and the Society of Apothecaries: First examination, Parts I., II., III., 35 guineas. First examination, Part IV., and second examination, 80 guineas, payable in two instalments of 40 guineas each. All departments in the Faculty of Medical Sciences are open to women students on the same terms as to men. The new Anatomy Building and the extension of the Physiology and Pharmacology Buildings, which have been provided by the benefaction of the Rockefeller Foundation of New York, and were opened by the King in May, will be in full use at the opening of the session in October.

### Charing Cross Hospital.

This hospital is fully organised for University teaching, and contains 300 beds. The courses of study are specially designed to meet the requirements of the University of London degrees, the Conjoint Board diplomas, and the final studies of the Universities of Oxford and Cambridge. Men and women students are admitted on equal terms. Fully-equipped laboratories are available for pathological and bacteriological work. Fees: Entrance fees—Primary and Intermediate, 10 guineas; final, 8 guineas. Annual fee, 40 guineas. The usual resident and students' appointments are open to students, and valuable prizes are awarded annually. Full information respecting the Medical School and the course of study recommended may be obtained on application to the Dean, Dr. W. J. Fenton, Charing Cross Hospital, Medical School, W.C. 2.

### Guy's Hospital.

The hospital contains 626 beds. Students are appointed to dresserships and clerkships in the wards and out-patient departments on the 16th day of October, January, April and July. Since 1904 the medical school buildings have been greatly extended. The Wills Library was presented in 1903, the Gordon Museum in 1905. A large extension was opened in July, 1923. This comprises new Departments of Anatomy, Biology, and Physics, and an extension of the Chemistry and Physiology Departments. The buildings and equipment are therefore thoroughly modern and give ample accommodation for the pre-clinical subjects. The College, within which are the premises of the Students' Club, affords residential accommodation for about 60 students. Fees and Courses.—First Year.—For Preliminary Science Course: £22 8s. for twelve months or less period, deducted from the entrance fee, payable as a second year's student. A special fee of £7 is charged for materials for this course. Second or Third Year (after first M.B.): Entrance fee, £28. Annual composition fee, £49, including all necessary materials. Fourth year (after second M.B.): Entrance fee, £14. Annual composition fee, £49, including all necessary materials. Provided a student has paid three annual composition fees, a proportionate rebate will be allowed from the last on his obtaining an approved qualification at any time within nine months of the last payment. Entrance scholarships to the value of £660 awarded annually in July. For further particulars, and permission to be conducted over the school buildings, application should be made to the Dean, Guy's Hospital, S.E. 1.

Guy's Hospital Dental School.

The present buildings were extended and rebuilt in 1912. They now form a block of buildings occupying four sides of a courtyard and consisting of three floors. The whole professional training of the dental student is carried out at Guy's. Beds are provided in the wards of the hospital for patients suffering from fractures and diseases of the jaw, and weekly demonstrations are given on surgical cases of interest to dental students. The following appointments are open to students every year without examination: Eighteen house surgeons, fifteen assistant house surgeons, fifteen clinical assistants, eight student demonstrators. Composition fees: Dental studentship, £70 for each of the four years; dental and medical studentship, £70 for the first three years; and eighty guineas for the fourth; and £56 each for the fifth and sixth. B.D.S. students: Preliminary Science subjects, £22 8s.; materials, £7; second, third and fourth years, £80 per annum; fifth year, £70.

King's College Hospital: University of London.

Situated at Denmark Hill, the hospital contains 400 beds. In 1922 there were 198,456 attendances in the casualty and out-patient departments. The hospital possesses every modern requirement for both patients and students. There is ample opportunity for any student who wishes to specialise in a subject to continue his studies with the help of junior staff appointments. The teaching of the preliminary and intermediate subjects is given at King's College in the Strand. Students come to the hospital for their advanced work, when their studies are co-ordinated under the direction of senior members of the honorary staff, assisted by medical, surgical and obstetric tutors. Fourteen resident appointments are made half-yearly. The composition fee for advanced medical studies is 93 guineas if paid in one instalment, or 95 guineas if paid in two instalments, in addition to the entrance fee of 10 guineas. The composition fee for the whole University or Conjoint course is 200 guineas. Two entrance scholarships (£50 each), for University students, will be offered for competition in September, 1923. Full particulars, and the school calendar, may be obtained from the Dean, Dr. H. Willoughby Lyle, or from the Secretary of the Medical School.

London Hospital.

The London Hospital is the largest institution of its kind in England, and contains 950 beds. During last year 17,444 in-patients and 107,455 out-patients received treatment, while 7,215 major operations were performed. Holders of resident appointments have free board. Special classes are held for the higher examinations of the University of London, the Royal College of Physicians, and the Royal College of Surgeons. Special entries for medical and surgical practice can be made. A residential hostel on hospital grounds is provided for the convenience of students. Scholarships and Prizes: Price Scholarship, £100, and one Entrance Scholarship, £50, subjects of First Medical Examination at the University of London; Epsom College Scholarship, free education, subjects of the First Medical Examination as above; Price Scholarship, open to students of Oxford and Cambridge Universities, £52 10s., human anatomy and physiology. Numerous prizes are offered in the various subjects of the curriculum. Medical Research Funds provide valuable scholarships for men wishing to undertake research or desirous of preparing theses for University degrees. Dean: Professor William Wright, London Hospital Medical College, E. 1.

London Hospital Dental School.

The school, which is fully equipped on the most modern lines and with the latest appliances, is an integral part of the college and hospital. A systematic course of instruction in dental prosthesis is arranged for pupils. The laboratory is in charge of a skilled curator and his assistants. Students in the school have the advantage of pursuing the medical portion of the dental curriculum in a college and hospital, of which the Dental School is part. Lecturers: Messrs. E. Sprawson, M.C., H. Chapman. H. D. Clapham, and A. Dinnis, Professor William Wright, Professor H. E. Roaf, Dr. Paul Fildes, Dr. R. J. Probyn-Williams, Messrs. H. Whyte, H. Candy; Director of Dental Studies: E. Sprawson, M.C., M.R.C.S., L.R.C.P., L.D.S.; Dean: Professor Wm. Wright, M.B., D.Sc., F.R.C.S.; Secretary: E. J. Burdon.

London (Royal Free Hospital) School for Women.

The Royal Free Hospital is in Gray's Inn Road, and the London School of Medicine for Women in Hunter Street, Brunswick Square, W.C. Under agreements the clinical practice of the Elizabeth Garrett Anderson Hospital, the Cancer Hospital, the National Hospital for Nervous Diseases, the Hospital for Sick Children, the South London Hospital for Women and Royal London Ophthalmic Hospital are also available for students of the school. Residence is provided in the Students' Chambers attached to the school. Numerous scholarships and prizes are offered annually in connection with the school, including the Isabel Thorne Scholarship of £30, the St. Dunstan's Medical Exhibition of £60 a year for three or five years, the Mabel Sharman-Crawford Scholarship of £20 a year for four years, the Mrs. George M. Smith Scholarship of £50 a year for three or five years, the Sir Owen Roberts Scholarship of £75 a year for four years, the Dr. Margaret Todd Scholarship of £37 10s. a year for four years, the Sarah Holborn Scholarship of £20 a year for three or five years, and the Bostock Scholarship of £90 for four years. Numerous resident appointments are available for students of the school after qualification. The session will begin on Monday, October 1st.

Middlesex Hospital.

Contains 455 beds. The cancer wing (containing 92 beds) and special investigation laboratories offer unrivalled opportunities for the study of cancer, both in its clinical and pathological aspects. There are also electro-therapeutic and electro-cardiographic departments. The Bland-Sutton Institute of Pathology contains a lecture theatre, large laboratories for pathological, bacteriological, and clinical investigation, and smaller rooms for original research work. Special courses of instruction are arranged twice yearly for the Diploma of Public Health. Eight registrars, six house physicians, eight house surgeons, two obstetric and gynæcological surgeons, two casualty medical officers, two casualty surgical officers, one resident anæsthetist, and two resident officers to the special departments are appointed annually. without extra fee of any kind. Non-resident qualified clinical assistants are appointed to assist in the various out-patient departments. Numerous scholarships, medals, and prizes, to the value of over £1,000, are open for competition among students of the school. The winter session will open on Tuesday, October 2, at 3 p.m. For prospectus and full particulars, apply to the Dean, Mortimer Street, W. 1.

St. Bartholomew's Hospital.

The oldest and second largest hospital in London. The medical college is complete in every department, and provides lectures and practical laboratory teaching in the preliminary sciences and intermediate subjects, as well as in the purely professional and clinical parts of the curriculum. Whole-time Directors of Medical and Surgical Clinics have been appointed, and all students receive a part of their training in these Clinics. Scholarships and prizes to the total value of nearly £1,000 are awarded annually.

St. George's Hospital.

Contains 336 beds, and has a convalescent hospital of 100 beds at Wimbledon. The usual resident and students' appointments are open to students. Scholarships and endowed prizes, amounting to £576, are awarded every year. The entire teaching and laboratories are now devoted to purely clinical subjects, to the great advantage of students in their fourth and fifth years of study. Arrangements have been made with the University of London for students who enter St. George's during the first, second, or third year of the curriculum to carry out the necessary courses of instruction at either University College or King's College. Students have therefore the advantages of the lectures and practical classes of these Colleges of the University during the preliminary and intermediate portions of their studies, and then complete their course in a school entirely devoted to medicine, surgery, and the study of disease. Further particulars may be obtained on application to the Dean of the Medical School, J. A. Torrens, M.D., F.R.C.P.

St. Mary's Hospital.

Contains 305 beds, and by a scheme of affiliation, for teaching purposes, of the Paddington Infirmary, Paddington Green Children's Hospital, and Maida Vale Hospital for Nervous Diseases, the teaching facilities extend over 1,000 beds. Clinical units in medicine and surgery were established in 1920, and have now been formally recognised by the University Grants Committee, St. Mary's being one of the six medical schools in London which enjoy this privilege. Lying-in beds have been added, and provide special facilities for the teaching of practical midwifery. Students also attend a short course of instruction at Queen Charlotte's Hospital before acting as maternity externs. There is an Institute of Pathology and Research, comprising seven special departments, the whole being under the personal direction of Sir Almroth Wright, F.R.S. Three Research Scholarships of £200 each are awarded annually to students working in the departments of the institute, and research beds have been opened. Clerkships in pathology, bacteriology and pathological chemistry, lasting for a period of three months, are open to students of the fifth year, and enable them to carry out the pathological and bacteriological investigations of the wards and learn the necessary technique under supervision. Seventy-two of these posts are available annually. The Medical School provides complete courses of instruction, and students can join at once on passing a preliminary examination in arts. Terms begin in October, January, and April. Two Open Scholarships of £210 each are awarded annually in July by nomination. The Palmer Scholarship (£25 per annum for two years) is similarly awarded in alternate years. One Scholarship of £200 is awarded annually, by nomination, to a student from a British or Overseas University. Fees: Composition fee for entire curriculum (5½ years), £200 in one sum, or £210 by five annual instalments; for clinical curriculum (2½ years), 90 guineas in one sum, or 95 guineas by two annual instalments.

### St. Thomas's Hospital.

This Hospital, with Medical School attached, contains 630 beds in constant use. The school buildings, which are separated from the Hospital by a quadrangle, comprise numerous theatres, laboratories and class rooms, well adapted for the modern teaching of large bodies of students in all subjects of the medical curriculum. There is a large library and reading room and a very complete museum. Five Entrance Scholarships are offered for competition annually —namely, in July, two scholarships in arts (matriculation subjects) giving free tuition for the first year of curriculum; two in science (chemistry, physics and biology) of £150 and £60 respectively); in September one of £100 open to University students who have passed anatomy and physiology for a medical degree in any of the British or Colonial Universities (subjects—anatomy, physiology, chemistry in its relation to medicine and physiology—any two). Numerous scholarships, prizes and medals are open for competition throughout the whole career of the student. Courses of instruction are provided for the Conjoint Diplomas, for the Preliminary, Intermediate and Final M.B. London, for Oxford and Cambridge Examinations, and for the Primary and Final F.R.C.S. Examinations. A resident assistant physician, a resident assistant surgeon, and a resident anæsthetist are appointed annually at a salary of £200 each per annum. Two hospital registrars (medical and surgical) at an annual salary of £250, an obstetric tutor and registrar (salary £50 per annum), an ophthalmic registrar (salary £50 per annum), and an orthopædic registrar (unpaid) are appointed yearly. Ten resident casualty officers and anæsthetists, seven house physicians, and nine house surgeons are appointed every six months. Clinical assistants in the special departments are appointed every three months, and hold office for six months if recommended for re-election. The fee for each year of study is £50. The annual fee covers all tutorial classes, but does not include instruction in infectious fevers, pharmacy, and vaccination. The prospectus of the school, containing further information, may be obtained on application to Dr. A. Elliot, Medical Secretary to the School, St. Thomas's Hospital, Albert Embankment, S.E. 1.

### University College Hospital.

Open to men and a limited number of women students who have passed a recognised examination in anatomy and physiology. The preliminary and intermediate studies may be carried out at University College or any other recognised medical school. Whole-time directors of medical and surgical units, with the aid of whole-time assistants, carry out the systematic training of the student in the principles of medicine and surgery, while the practical side is mainly dealt with by the honorary staff of the hospital. Scholarships, exhibitions and prizes to the value of over £1,000 are offered for competition every year. Composition fees for the final M.B., B.S. course of the University of London and for the medical education required by the Examining Board in England and the Society of Apothecaries, for the course required for the third examination, 112 guineas, or in two instalments of 70 and 45 guineas. The Dental School, Great Portland Street, W. (formerly the National Dental Hospital), has been recently reorganised and equipped with the most modern appliances for the teaching of dental surgery. The complete dental curriculum can be carried out at University College and University College Hospital. Particulars of the fees for the dental curriculum can be obtained from the Sub-Dean for dental students at the Dental School, Great Portland Street.

### Westminster Hospital.

Westminster Hospital affords relief to upwards of 2,600 in-patients and about 25,000 out-patients annually. There is a Students' Clubs Union, the subscription for membership of which is included in the general fees. The annual composition fee is 35 guineas. Women students are admitted. Five Entrance Scholarships are offered for competition amongst students entering in October, and two for those entering in May. Further particulars can be obtained from the Dean, Dr. A. S. Woodwark.

### PROVINCIAL MEDICAL SCHOOLS.

### Birmingham University.

In order to obtain the degrees of M.B. and Ch.B. four examinations have to be passed after passing Matriculation and Pre-Medical Examination in Chemistry and Physics. The University also grants degrees and a diploma in dental surgery, and a diploma (D.P.H.) and a degree in Public Health (B.Sc.). The General Hospital and the Queen's Hospital, containing between them over 600 beds, are amalgamated for the purposes of clinical instruction, which is carried on under the direction of the University Clinical Board. Medical students also have free access to clinical instruction at the Eye, Ear and Throat, Orthopædic and Spinal, Women's, and Children's Hospitals, which are associated with the University. The composition fee for the whole course of lectures and instruction at the University is £106 5s., besides which there is a composition fee for attendance on the medical and surgical practice and the clinical lectures at both the General and Queen's Hospitals of £52 10s., making a total of £158 15s. for courses at University, and General and Queen's Hospitals for M.B., Ch.B. The total cost of the five years' curriculum, including all courses required, membership fees and examination fees for M.B., Ch.B., is £197 0s. 6d. Dean: Mr. William F. Haslam, F.R.C.S. The winter session opens on October 1.

### Bristol University.

Grants the conjoined degrees of M.B., Ch.B., the degrees of Doctor of Medicine (M.D.), Master of Surgery (Ch.M.), Bachelor of Dental Surgery (B.D.S.), and Master of Dental Surgery (M.D.S.), also diplomas in Public Health (D.P.H.), in Dental Surgery (L.D.S.), and in Veterinary State Medicine (D.V.S.M.). For the conjoined degrees of M.B., Ch.B., the curriculum occupies five years after passing Chemistry and Physics of the first examination. The University lectures and laboratory courses are attended in the large and well-equipped new wing of the University. Hospital practice and clinical instruction are provided in the allied hospitals, which together afford upwards of 600 beds and a very large external midwifery department. Three years at least must be spent in the University, and two of them subsequently to passing the second examination. Women are admitted to all degrees and diplomas and to the courses of study necessary for them. The winter session opens on October 2.

### Cardiff.

Students of the Welsh National School of Medicine, University College, Cardiff, can complete the whole of their curriculum in the school. The courses of instruction qualify for the degrees in Medicine and Surgery of the University of Wales (which see), and certain of them also for the degrees of other Universities and for the diploma of the Conjoint Board. Clinical instruction is given at the Cardiff Royal Infirmary and at other local hospitals. The Cardiff Royal Infirmary contains between 300 and 400 beds. It is well equipped in all general and special departments, giving facilities for every branch of study. All classes are open to both men and women students. Medical practitioners wishing to prepare for the Diploma in Public Health or the Tuberculous Diseases Diploma of the University of Wales can attend complete courses of instruction in the school. Prospectuses can be obtained on application to the Dean of the Faculty of Medicine or from the Secretary of the School, D. J. A. Brown, University College, Cardiff. The next Session opens on October 2.

### Durham University.

For students who intend to graduate at Durham University it is necessary that one of the five years of professional education should be spent in attendance at the College of Medicine, Newcastle-upon-Tyne. During the year the student must attend lectures at the College and the medical and surgical practice and clinical lectures at the Royal Victoria Infirmary, which contains about 550 beds. The remaining four years of the curriculum may be spent at Newcastle-upon-Tyne or at one or more of the recognised medical schools. There are four professional examinations for the M.B., B.S. degrees. Particulars as to fees, etc., may be obtained from Professor Howden, Registrar, College of Medicine, Newcastle-upon-Tyne.

### Leeds University.

Grants the degrees of M.B. and Ch.B., M.D. and Ch.M., M.Ch.D. and B.Ch.D., and, in association with the Universities of Liverpool, Manchester, Sheffield, and Birmingham, holds a Matriculation examination. Diplomas in Public Health, Psychological Medicine and Dental Surgery are also granted. Clinical instruction is given in the General Infirmary, which contains 632 beds, and is amply provided with special departments. Students of the University also have access for instruction to Leeds Public Dispensary, the City Fever Hospitals, the Hospital for Women and Children, the Leeds Maternity Hospital and the West Riding Asylum at Wakefield.

### Liverpool University (Faculty of Medicine).

Grants degrees in Medicine (M.B.; M.D.), in Surgery (Ch.B.; Ch.M.; M.Ch. Orth.), and in Hygiene (M.H.). Candidates for degrees are required to pass the Matriculation examination or an equivalent. Students may study in the University for the degrees or for the examinations of other licensing bodies. Courses of instruction in tropical diseases can be obtained at the Liverpool School of Tropical Medicine. Fellowships, scholarships, and prizes of over £1,500 are awarded annually, in addition to entrance scholarships. The Clinical School of the University now consists of four general hospitals—the Royal Infirmary, the David Lewis Northern Hospital, the Royal Southern Hospital, and the Stanley Hospital; and of six special hospitals—the Eye and Ear Infirmary, the Hospital for Women, the Liverpool Ladies' Charity and Maternity Hospital, the Royal Liverpool Children's Hospital, St. Paul's Eye Hospital, and St. George's Hospital for Skin Diseases. These hospitals contain in all about 1,140 beds. Their organisation to form one teaching institution provides the medical student and the medical practitioner with a field for clinical education and study unrivalled in extent in the United Kingdom. The University also grants a licence and degrees of Bachelor and Master in dental surgery, a diploma and degrees of Bachelor, Master, and Doctor in veterinary medicine and surgery, a diploma in public health, a diploma in tropical medicine, and a diploma in medical radiology and electrology. The Dental School and Dental Hospital are well equipped and provide complete instruction in the subjects for the degrees and diplomas conferred by the University and examining bodies.

### Manchester, Victoria University.

The medical department of the University, which is open to both men and women students, is provided with facilities for instruction in all the courses for degrees and the professional qualification and for teaching the preliminary subjects. Clinical instruction is provided at the Manchester Royal Infirmary, which contains 592 beds, and also has large out-patient and casualty departments. Associated with the Infirmary are a Convalescent Hospital at Cheadle, containing 136 beds, and the Royal Lunatic Hospital, accommodating 430 patients. Clinical instruction in obstetrics and gynæcology is also given at St. Mary's Hospital and other hospitals.

### Sheffield University (Faculty of Medicine).

The degrees of the University, M.B., Ch.B., M.D., Ch.M., and the diploma in dental surgery are open to men and women alike. A candidate for the degrees of M.B., Ch.B., must produce certificates that he will have attained the age of 22 on graduation; that he has pursued the courses of study required by the University Regulations during a period of not less than five years subsequently to the date of his matriculation or exemption from matriculation and to the date of passing the further examination in Chemistry and Physics, three of such years at least having been passed in the University, one at least being subsequent to the passing of the first examination. The composition fee for University lectures, practical classes and hospital practice is £38 a year for each of the five years. The University of Sheffield offers many valuable scholarships and prizes to students in the Faculty of Medicine. Dental Department.—The University awards degrees (B.D.S. and M.D.S.) and a diploma (L.D.S.) in Dental Surgery. Candidates for the degree of B.D.S. and the diploma of L.D.S. must before entering upon the course either pass or obtain exemption from the Joint Board Matriculation examination and pass the further examination in Chemistry and Physics. The course for the B.D.S. extends over a period of five and a half academic years and that for the L.D.S. over a period of four years. The tuition fees for the courses are £25 per annum and a further £50 per annum payable each year the student receives instruction in Mechanical Dentistry in the Dental Department.

### Wales (University of).

Grants degrees in medicine, diploma in public health, and a tuberculous diseases diploma. At the four constituent colleges of Aberystwyth, Bangor, Cardiff and Swansea there are departments of chemistry, botany, zoology and physics, so that students can obtain proper instruction in the preliminary subjects. A National School of Medicine has been established in connection with University College, Cardiff (which see).

### SCOTTISH MEDICAL SCHOOLS.

### University of Edinburgh.

Four degrees in medicine and surgery are conferred, namely, Bachelor of Medicine (M.B.), Bachelor of Surgery (Ch.B.); Doctor of Medicine (M.D.), and Master of Surgery (Ch.M.). The degree of Ch.B. is not conferred on any person who does not at the same time obtain the degree of M.B., and the degree of M.B. is not conferred on any person who does not at the same time obtain the degree of Ch.B. To obtain the medical degree it is necessary to pass the preliminary examination or its recognised equivalent, and to pass four professional examinations. To obtain the M.B., Ch.B. degrees, it is necessary to spend at least two of the five years of medical study in the University of Edinburgh. Clinical instruction is given in the Royal Infirmary, which contains nearly 900 beds; Royal Hospital for Sick Children, with 120 beds; Maternity Hospital, with 40 beds available for clinical instruction; City Fever Hospital, with 600 beds; and the Royal Mental Hospital, Morningside, with 500 beds. The total number of beds available for the clinical instruction of students of the University is 2,760. The fees for the four professional examinations amount to £34 13s. Courses of instruction are given for the degrees of B.Sc. and D.Sc. in Public Health and for the diplomas in Public Health, Tropical Medicine and Hygiene, and Psychiatry. These diplomas are open to approved registered practitioners, as well as to graduates in medicine and surgery of the University. The University also takes part in the courses given under the auspices of the Edinburgh Post-Graduate Courses in Medicine. In the departments of the Faculty of Medicine provision is made for research by students of graduate standing. In the laboratories facilities will be provided for candidates for the degree of Ph.D. whose applications to engage in research have been accepted by the Senatus. Communications should be addressed to the Dean of the Medical Faculty, University New Buildings, Edinburgh.

School of Medicine of the Royal Colleges (Edinburgh).

This school is constituted by an association of lecturers who lecture in several buildings near the Royal Infirmary. The lecturers are recognised or licensed by the Royal Colleges of Surgeons and Physicians, and also by the University. The teaching is similar to that of the Scottish Universities, and the students receive similar certificates at the close of each session. The lectures qualify for the University of Edinburgh and other Universities; the Royal College of Physicians and Surgeons of Edinburgh, London and Dublin; the Royal Faculty of Physicians and Surgeons of Glasgow and other Medical Boards. The whole education required for graduation at the University of London may be taken in this school. The laboratories open and the lectures commence on October 9. The facilities for clinical instruction are similar to those provided for the University. Full particulars and a copy of the calendar of the school may be had (price 6d.) from the Dean of the School, 11 Bristol Place, Edinburgh.

University of Glasgow.

The whole course of study for the M.B., Ch.B. can be passed in the Medical School of the University. The regulations are similar to those stated for Edinburgh. Clinical instruction is given at the Western Infirmary, which contains about 600 beds; at the Royal Infirmary, which contains over 600 beds; and at the Eye Infirmary, the Maternity Hospital, the City Fever Hospitals and various other hospitals. The cost of the course, including matriculation, fees for classes, for hospital attendance, and for professional examinations, amounts to about £250.

Anderson College of Medicine.

The college, which provides both Medical and Dental curricula, is situated in Dumbarton Road, Glasgow, adjoining the Western Infirmary and the University. Degrees and diplomas: Certificates of attendance on the lectures are accepted by the Universities of London and Durham, by the Universities of Glasgow, Edinburgh, &c., under conditions stated in the calendars, and by all the Royal Colleges and licensing boards in the United Kingdom. The public-health course qualifies for the Scottish Licensing Board, the Irish Colleges and the Universities of Oxford, Cambridge, London, &c. Courses of instruction for intending Probationer Nurses will be held in the scientific subjects which are embraced in the Syllabus of the General Nursing Council for Scotland. This preliminary course, it is expected, will greatly ease the strain on the Probationer Nurse during the subsequent period of hospital training. There will be three courses of lectures during the year. The Carnegie Trust extends its benefactions to students of Anderson's College. A calendar will be sent on receipt of a postcard by the Secretary to the Medical Faculty, The Anderson College of Medicine, Glasgow, W.

Queen Margaret College (Women's Department of the University).

This is an integral part of the University of Glasgow. The courses, regulations and fees are the same as for men. The instruction is given by University professors and lecturers appointed by the University Court, partly in mixed and partly in separate classes. The College provides a separate building, with classrooms, laboratories, library, and other teaching appliances. The women have all the rights and privileges of University students. Clinical instruction is provided in the Royal Infirmary, Western Infirmary, Victoria Infirmary, and in other hospitals. Students who desire to begin the study of medicine in April, 1924, should apply for the necessary forms to the Mistress of Queen Margaret College, before the end of January, 1924. The number of women students in he Faculty of Medicine during 1922-23 was 318.

St. Mungo's College (Glasgow Royal Infirmary).

The college buildings are situated within the grounds of the infirmary, and are provided with classroom and laboratory accommodation for several hundred students. Students receive their clinical instruction in the wards of the infirmary, which contains 615 beds, and in the Glasgow Royal Maternity and Women's Hospital. The college provides a complete medical curriculum, and the classes qualify for the diplomas of the English, Scottish and Irish Conjoint Boards, and under certain conditions for the various Universities. Students who have fulfilled the conditions of the Carnegie Trust are eligible for the benefits of this Trust during the whole course of their studies at St. Mungo's College. The classes are open to male and female students equally. Students entering for the Dental diploma can take all their surgical and medical classes at St. Mungo's College. There is a special department of public health, attendance on which qualifies for the Conjoint Board and the Universities of Oxford and Cambridge. The inclusive fees for the whole medical curriculum amount to about £120. The syllabus may be obtained on application to the Secretary to the Faculty of Medicine, 86 Castle Street, Glasgow.

University of Aberdeen.

The Faculty of Medicine embraces twelve chairs and numerous readerships and lectureships in the various branches of medicine and surgery. There are fully equipped laboratories and museums. The degrees of M.B. and Ch.B. are conferred together; they cannot be obtained separately. The curriculum of study extends over a period of not less than five years. An inclusive fee of 126 guineas is payable for instruction within the University, and the fee for the Degrees of M.B. and Ch.B. is 33 guineas, payable in four instalments. The total cost of the curriculum, including hospital fees, class and matriculation fees and degree fee, is approximately £236. Besides the Royal Infirmary, students have the opportunity of attending several other local institutions where complete courses of clinical instruction are given. Bursaries, scholarships and fellowships to the number of fifty, and of the annual value of over £1,180, are open to students of medicine. The Degree of Doctor of Medicine may be conferred on a qualified candidate who has obtained the degrees of M.B. and Ch.B. of the University. The candidate must present a thesis for approval by the medical faculty and pass a clinical examination in medicine or a special department of medical science. Similar regulations apply to the degree of Ch.M. (Master of Surgery). The degree of Doctor of Philosophy (Ph.D.) is also granted in the Faculty of Medicine. A Diploma in Public Health is conferred, after examination, on graduates in medicine of any University of the United Kingdom.

University of St. Andrew's.

Medical students may attend for the first two years of study in St. Andrews (or in Dundee). The remainder of the course must be taken in Dundee, and full particulars of the curriculum and examinations may be obtained from the Dean of the Faculty of Medicine, the Medical School, Dundee. Clinical instruction is given at the Dundee Royal Infirmary, which has 400 beds (including Maternity Hospital), at the Dundee Eye Institution, the King's Cross Fever Hospital, and the Dundee District Asylum.

Carnegie Trust and the Payment of Class Fees.

This is a fund to provide under certain regulations eligible students with all or a portion of the class fees of the curriculum. Applicants must be over 16 years of age, of Scottish birth or extraction, and must have obtained the Leaving Certificate of the Scottish Education Department or recognised equivalent. Forms of application are to be obtained from the Secretary, Carnegie Trust for the Universities of Scotland, 22, Hanover Street, Edinburgh.

### IRELAND.

Belfast (Queen's University).

Provides all the classes required for a complete medical curriculum. Women are eligible as students on the same terms as men. Clinical instruction is given at the Royal Victoria Hospital, which was rebuilt a few years ago and has 300 beds, and at the Mater Infirmorum Hospital, which has 150 beds. Various other hospitals are open to students. The cost of the curriculum intended for students proceeding to the degrees of the University is approximately £130. This includes examination fees and a perpetual ticket for attendance at the Royal Victoria Hospital or the Mater Infirmorum Hospital and fees for the special hospitals. The course for the Conjoint Board costs about the same amount. The matriculation regulations and full information regarding entrance and other scholarships can be obtained from the Secretary, Queen's University, Belfast. The calendar is published in September, price 2s. There is a Department of Dentistry in which students are prepared for the L.D.S., B.D.S., and M.D.S.

